# The Association of Metabolic Syndrome and Urolithiasis

**DOI:** 10.1155/2015/570674

**Published:** 2015-03-22

**Authors:** Yee V. Wong, Paul Cook, Bhaskar K. Somani

**Affiliations:** ^1^Department of Urology, University Hospital Southampton NHS Trust, Southampton SO16 6YD, UK; ^2^Department of Biochemical Pathology, University Hospital Southampton NHS Trust, Southampton SO16 6YD, UK

## Abstract

There has been an increasing prevalence of kidney stones over the last 2 decades worldwide. Many studies have indicated a possible association between metabolic syndrome and kidney stone disease, particularly in overweight and obese patients. Many different definitions of metabolic syndrome have been suggested by various organizations, although the definition by the International Diabetes Federation (IDF) is universally considered as the most acceptable definition. The IDF definition revolves around 4 core components: obesity, dyslipidemia, hypertension, and diabetes mellitus. Several hypotheses have been proposed to explain the pathophysiology of urolithiasis resulting from metabolic syndrome, amongst which are the insulin resistance and Randall's plaque hypothesis. Similarly the pathophysiology of calcium and uric acid stone formation has been investigated to determine a connection between the two conditions. Studies have found many factors contributing to urolithiasis in patients suffering from metabolic syndrome, out of which obesity, overweight, and sedentary lifestyles have been identified as major etiological factors. Primary and secondary prevention methods therefore tend to revolve mainly around lifestyle improvements, including dietary and other preventive measures.

## 1. Introduction

The prevalence of urolithiasis is increasing globally and is observed across sex, age, and race [1]. Although the prevalence is between 5 and 10% in Europe, it is as high as 20% in some wealthy countries such as Saudi Arabia. Also, the recurrence rates are variable and can be up to 50–75% within 10 years. In the United Kingdom, there has been a 63% increase in nephrolithiasis episodes and a 127% increase in ureteroscopic stone treatments in the past ten years [2]. With a rise in the incidence of stone disease and the surgical intervention for it, there is a significant cost to the patients and the economy.

Many studies have suggested that obesity is a significant contributing factor to urolithiasis with the World Health Organisation (WHO) estimating that 1.7 billion people are overweight and obese worldwide [3]. An increased incidence of urolithiasis of greater than 75% is seen in overweight and obese patients compared to their normal counterparts [4]. The increased incidence and prevalence of urolithiasis are in parallel with the rising incidence of metabolic syndrome. The latter leads to a rise of uric acid stones, increase in urinary phosphate and oxalate levels, and an overall rise in the ratio of stone disease in females. In addition there seems to be a positive association between obesity and the risk of first time and recurrent stone formation with a decreased time to recurrence in obese patients compared to the normal population. In USA, the treatment of urolithiasis caused by obesity and diabetes is estimated to cost 1.24 billion US dollars per year by the year of 2030 [4]. This review article will discuss the different definitions of metabolic syndrome, the proposed pathophysiology, the etiology, and the preventative measures for urolithiasis with metabolic syndrome.

## 2. The Definition of Metabolic Syndrome

Various definitions of metabolic syndrome have been proposed by expert groups since 1998. However, their different sets of conflicting and incomplete diagnostic criteria have been seen as a controversial issue. The American Heart Association (AHA) [5], the World Health Organization (WHO) [6], the European Group for the Study of Insulin Resistance (EGIR) [6], and the National Cholesterol Education Program—Third Adult Treatment Panel (NCEP ATP III) [7] have similarities on the core components of metabolic syndrome: central obesity, dyslipidemia, hypertension, and insulin resistance [8]. However, the WHO diagnostic criteria of metabolic syndrome require the presence of diabetes mellitus, impaired glucose tolerance, impaired fasting glucose, or insulin resistance, along with any 2 (or more) of the core components, for a patient to be considered as suffering from metabolic syndrome. Similarly, EGIR requires insulin resistance along with two of the other core components, for a patient to be considered as suffering from metabolic syndrome. While the threshold values on the core components for clinical diagnosis of metabolic syndrome are similar between the different classifications, the exact value for each component does, however, vary slightly between the different classifications (see Table 1).

The International Diabetes Federation (IDF) recognised the need for a single, universally accepted diagnostic tool for metabolic syndrome. The new IDF definition of metabolic syndrome differs from the other definitions in that it requires the evidence of central obesity defined by waist circumference (WC) with ethnicity specific threshold values (see Table 2) and two or more of the other core components. However, if body mass index (BMI) is more than 30 kg/m^2^, central obesity may be assumed and waist circumference does not need to be measured. The IDF consensus group has included a few further parameters that have been found to correlate with metabolic syndrome; these should be included in future research studies. These additional diagnostic parameters are abnormal body fat distribution, atherogenic dyslipidemia, dysglycemia, insulin resistance, vascular dysregulation, proinflammatory state, prothrombin state, and hormonal factors and may be expected to further modify the diagnostics for metabolic syndrome in relation to different ethnic groups [9].

## 3. Proposed Pathophysiology of Urolithiasis in Metabolic Syndrome

The formation mechanism of urolithiasis is very complex; currently no single pathophysiological theory can properly account for urolithiasis formation with metabolic syndrome. Over the past decade, many studies have been conducted on the topic of pathophysiology for urolithiasis with metabolic syndrome and several hypotheses have been proposed including hypocitraturia, hyperuricosuria, hyperoxaluria, and hypercalciuria. An independent association of urolithiasis with all traits of metabolic syndrome was noted including diabetes, cardiovascular disease, hypertension, and dyslipidemia.

### 3.1. Insulin Resistance

Insulin resistance has been shown to have causal links between metabolic syndrome and lithogenesis. Insulin resistance decreases the production and transport of ammonia, resulting in changes in urine acidification and lowered urine pH [10]. The alteration in urine acidification may be due to defective apical membrane H^+^ and NH^+^_4_ secretion whereas the lowered urine pH may be caused both through defective ammonia synthesis by the proximal tubule cell and through ammonium transport into the renal tubular lumen [11]. 

**Figure 1:**
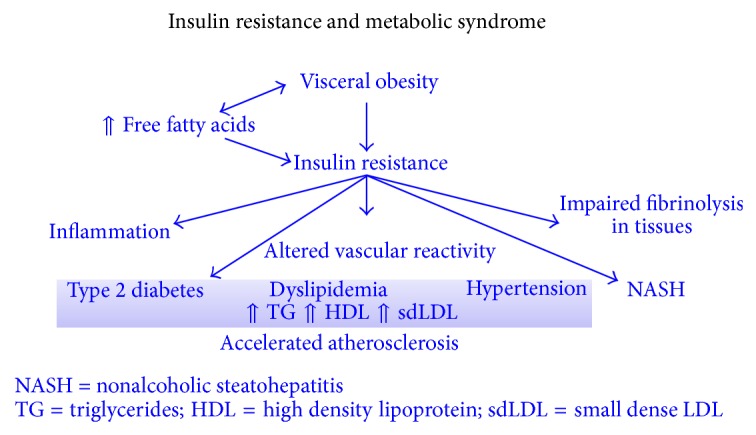
Pathophysiology of insulin resistance in metabolic syndrome [56].

Free fatty acids (FFA) are released from adipose tissue due to visceral obesity. FFA increase the production of glucose, triglycerides, very low density lipoprotein (VLDL), and low density lipoprotein (LDL) and decrease the production of high density lipoprotein (HDL) in the liver, resulting in type II diabetes and dyslipidemia. Furthermore they reduce insulin sensitivity by inhibiting insulin-mediated glucose uptake. The resulting increase of circulating glucose results in inflammation, altered vascular reactivity, and impaired fibrinolysis in tissues (see Figure 1).

### 3.2. Randall's Plaque Hypothesis

Urine crystals can be attached to an exposed site of interstitial calcium phosphate as a consequence of injury to the renal papilla, otherwise known as Randall's plaque. The mechanism of interstitial plaque formation has not been fully understood eight decades after Alexander Randall formed his hypothesis, as his work was carried out in cadaveric kidneys instead of a targeted stone-forming population [12]. However, Randall's plaque theory has been amplified in recent years due to endoscopic visualisation of calcium phosphate deposits at the tip of renal papillae, the origin of renal calculi [13]. A study has shown that Randall's plaque is frequently found in calcium oxalate stone formers, thus illustrating the importance of the hypothesis in the pathogenesis of calcium oxalate urolithiasis [14].

**Figure 2:**
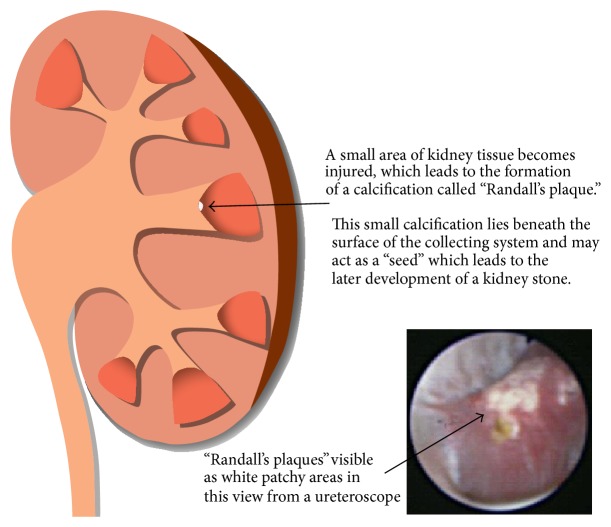
Description of Randall's plaque and the view from a ureteroscope [57].

Randall's plaque is primarily found on the thin basement membrane at the beginning of the loop of Henle which is believed to be the primary site of stone attachment [15]. Evan illustrated, in an endoscopic and histologic study, that the majority of calcium oxalate stones (75%) are formed attached to sites of Randall's plaque in idiopathic calcium oxalate stone formers [16]. Furthermore, a unified theory on pathogenesis of Randall's plaque was proposed where urinary abnormalities such as hypercalciuria, hyperoxaluria, hypocitraturia, renal stress, or trauma lead to renal epithelial injury. The nucleation of calcium phosphate crystals is then enhanced by an increased production of bone-specific proteins and reduced inhibition of crystallization inhibitors, leading to calcification of membranous cellular degradation products and other fibers until the plaque reaches the papillary epithelium (see Figure 2) [17]. The renal epithelium exposure to crystals of calcium phosphate generates reactive oxygen species (ROS), causing injury and the development of oxidation stress [18]. Randall's plaque has recently become a new prognostic factor for idiopathic calcium urolithiasis and an indicator for stone recurrence by endoscopic assessment of the papillary calcification [19].

### 3.3. Pathophysiology of Uric Acid Stones

**Figure 3:**
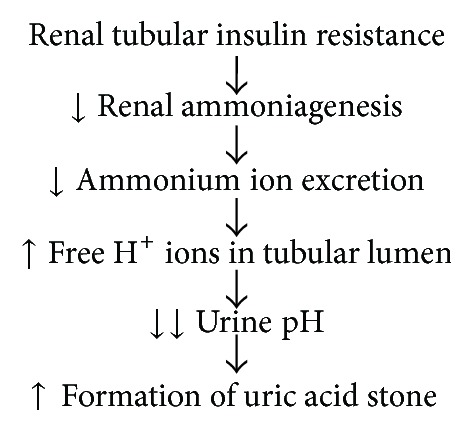
Pathophysiology of uric acid stone formation in metabolic syndrome patients [20].

In their paper, Sakhaee and Maalouf revealed two major abnormalities that were found to result in abnormally acidic urine: an increased acid excretion and a decreased ammonium excretion [20]. Amongst the risk factors for uric acid stone formation are acidic urine, hyperuricosuria, and low urinary volume, but these may stem from genetic, secondary, or idiopathic causes [21]. Increased uric acid excretion appears to be commonly related to insulin resistance, which further relates with obesity [22]. However, while common, insulin resistance with undue acidity can only be observed in about two-thirds of affected patients. Increased uric acid excretion on its own is generally not sufficient to detrimentally increase urinary acidity, although it does take part in a larger process to that effect. Defective buffering of ammonium due to decreased renal ammonium production, however, further lowers the urinary pH, resulting in the formation of uric acid stone (see Figure 3) [23].

In the absence of hyperuricosuria, a decrease in the urinary pH (pH < 5.5) can lead to the formation of less soluble uric acid from urinary urate, leading to uric acid stones. A low urine pH may be seen in conditions such as chronic diarrhoea or from excessive acid load. In addition, a close link can be observed between uric acid stones formation and diabetes; type II diabetes appears to be common in uric acid stone formers and conversely rate of uric acid stones in diabetics is around 30–40% compared to a rate of 5–10% in the general population [23]. In addition to insulin resistance, other parameters of metabolic syndrome including overweight, arterial hypertension, and diabetes mellitus are risk factors for uric acid stone formation [10]. Gout and myeloproliferative disorders are current controversial issues associated with uric acid stones but the pathophysiology remains unclear [24].

### 3.4. Pathophysiology of Calcium Stones

The pathophysiological mechanism of calcium stones is more complex than that of uric acid stones; the mechanism includes low urine volume, hypercalciuria, hyperuricosuria, hypocitraturia, hyperoxaluria, and the abnormalities in urine pH [25]. Out of these, hypercalciuria has been found to be the most significant pathophysiological factor in calcium lithogenesis [26]. This can be from the kidney due to renal leak, or from bone resorption or from absorption from the gastrointestinal (GI) tract. An increase in the GI calcium absorption is more common, usually associated with idiopathic osteoporosis from bone resorption.

Elevated sodium intake can also lead to crystallisation of calcium salts causing hypercalciuria and reducing levels of citrate that serve as inhibitors of urolithiasis. Hypercalciuria can also be caused by systemic acidosis and protein overload [27]. Furthermore a systemic imbalance between the essential fatty acid (EFA) omega-3 and the omega-6 pathways causes a higher phospholipid AA level responsible for hypercalciuria and hyperoxaluria [28]. Hyperoxaluria indirectly raises the urinary saturation of calcium oxalate and can lead to a higher risk of calcium containing stones [29]. The condition can arise from either oxalate-rich foods or conditions that increase the intestinal absorption and urinary excretion of oxalate, such as inflammatory bowel disease or bowel resection.

A study on the topic has similarly observed association between insulin resistance and calcium stone formation. Cupisti and colleagues estimated insulin resistance by the homeostatic model assessment-insulin resistance (HOMA-IR) value finding association between HOMA-IR value and lowered citrate excretion in calcium stone formers [29]. Citrate in the urine interacts with calcium particles to form soluble compounds and thus preventing calcium crystal growth in the urine. Hence, hypocitraturia is present in almost 20–60% of patients with calcium stones. Matlaga et al. demonstrated the role of Randall's plaques in the pathogenesis of calcium stones [30]. These plaques were calcium-containing deposits found in almost a quarter of renal papillae in autopsy studies.

Calcium phosphate stone formation differs from calcium oxalate stone formation, with the former resulting in apatite crystals depositing in the inner medullary collecting ducts with associated interstitial scarring. The abnormal reabsorption of bicarbonate in the thick ascending limb (TAL) of the loop of Henle results in higher urine pH in relation to the calcium phosphate stone formation [31].

## 4. Etiology of Urolithiasis in Metabolic Syndrome

Metabolic syndrome, a cluster of cardiometabolic alterations, is a multifactorial disorder leading to an increase of urinary acid secretion, thereby lowering the urine pH leading to acid crystal deposition and stones.

### 4.1. Overweight and Obesity

Abdominal obesity is known to be the most prevalent manifestation of metabolic syndrome [32]. It is associated with a complex neuroendocrine alteration in the body, where it causes insulin resistance with a combination of hypercortisolemia and deficiency of growth hormones, consequently escalating symptoms of metabolic syndrome [33]. Many studies have demonstrated that obesity and weight gain correlate with increased risk of kidney stone disease, where the increase in risk tends to be greater for women than for men [34]. An increased incidence of urolithiasis of up to 75% has been seen in patients who are overweight or obese [29]. Other contributing modifiable factors include a low fluid intake and dietary calcium with an excess of sodium and animal protein. Other than obesity, other metabolic traits such as diabetes mellitus, dyslipidemia, and hypertension increase the severity of kidney stone disease; Kohjimoto et al. indeed found that patients with 4 metabolic traits have 1.8 times larger odds of recurrent formation of stones as well as the formation of multiple stones compared to patients with 0 traits [35]. Dyslipidemia and hypertension have been associated with urolithiasis with more than a casual association noted in studies [29] but they, along with diabetes and obesity, form the four characteristic traits of metabolic syndrome. A higher prevalence of kidney stones has been observed with increasing number of these traits (up to 10% with all 5 traits) with the odds ratio showing up to a twofold increase. Lastly, Wrobel et al. showed an association of obesity and calcium oxalate stone disease with significant results in BMI, urine pH, and urine citrate, but obesity is not a predictor for the recurrence risk of calcium oxalate stone [36].

### 4.2. Genetics

Some people are genetically predisposed to developing insulin resistance, which can lead to metabolic syndrome. A few variants of metabolic syndrome genome have been identified, where they have been reported localising around lipid metabolism regulating genes. However there is no evidence for common genetic basis for clustering of metabolic syndrome traits [37]. Due to the complexity of the metabolic syndrome phenotype, there is still no explanation to the pathogenic mechanism of metabolic abnormalities [38].

A recent finding stated that sleep deprivation, shift work, and bright light exposure at night are causes with tendencies towards developing a phenotype resembling metabolic syndrome as well as increased adiposity [39]. The human genome has however not changed remarkably in the last decade, in the same period during which the prevalence of the metabolic syndrome has been increasing dramatically, thus representing the importance of gene-environmental interactions. Indeed there is good evidence that nutrition plays an important role in the development and progression of the metabolic syndrome [40].

### 4.3. Age and Sex

The prevalence of renal stone disease varies between 7–15% in males and 3–6% in females as the latter seem to be protected due to high urinary citrate which serves as an inhibitor of stone formation [19]. Oestrogen also seems to be protective by lowering urinary supersaturation of salts.

Hildrum et al. investigated the age-prevalence of metabolic syndrome according to the IDF definition of metabolic syndrome. The study observed an increase from 11.0% to 47.2% between males in the age groups of 20–29 and 80–89 years, respectively. A similar increase from 9.2% to 64.4% was observed for females in the same age groups. Therefore, a conclusion could be made where the prevalence of metabolic syndrome increases greatly with age [41]. Another study showed that rates of metabolic syndrome were two to three times higher in 50 years and older than in the younger age groups. This may be related to the medications used by older age groups which are a risk factor for metabolic syndrome [42].

### 4.4. Lifestyle

Similar to how metabolic syndrome can be strongly associated with abdominal obesity, metabolic syndrome can also be related to lifestyle, in particular a person's diet and physical activities. Consumption of high fat diets of course strongly correlates with obesity, which correspondingly worsens insulin resistance and other metabolic abnormalities [43]. A study suggested that consumption of Western dietary patterns, meats, and fried foods promotes the incidence of metabolic syndrome, whereas dairy consumption seems to provide some protection [44].

Dehydration (caused by warm climate or otherewise) contributes to urolithiasis. Due to a low urinary volume and high urine osmolality, there is increased urinary calcium and oxalate [19]. Similarly working in hot and humid conditions encourages the formation of renal stones [19]. Furthermore, physical inactivity is an important factor for the prevalence of metabolic syndrome. The National Health and Nutrition Examination Survey (NHANES) 1999 to 2000 reported that participants who did not engage in any physical activity during leisure time had almost twice the odds of having metabolic syndrome with odds ratio of 1.90 and 95% confidence interval (CI) of 1.22 to 2.97 compared to those who were reportedly engaged in ≥150 minutes of physical activity each week. [45].

## 5. Preventative Measures of Urolithiasis in Metabolic Syndrome

Due to a high risk of prevalence and recurrence of urolithiasis, modifiable lithogenic risk factors can be targeted to offer an inexpensive and convenient means to decrease it. As a vast number of these risk factors can be modified by dietary and environmental modifications, they have a pivotal role in the treatment.

### 5.1. Primary Prevention

The approach to prevention of urolithiasis rests on the reduction of lithogenic risk factors. A patient work-up with serum and 24-hour urine test might be necessary to identify the potential causative factors with necessary strategy to correct them. Patient education and promotion of healthy lifestyle are crucial to preventing metabolic syndrome. Pharmacists on pharmaceutical level should be able to provide more information on the topic and to create awareness about metabolic syndrome to the target population. Information provided should include the risk factors for metabolic syndrome, the complications of metabolic syndrome, the benefits of lifestyle changes for the prevention of (or treatment for) metabolic syndrome, and available weight control programmes [46]. Clinical practice guidelines set by the Endocrine Society have recommended regular screening and identification of patients at risk for metabolic syndrome with measurement of BMI or waist circumference, blood pressure, and glucose and lipid profile. Patients who are identified to be at risk should then undergo 10-year global risk assessment for follow-up [47].

The current dietary recommendation of fruit and vegetables intake acts as a preventative measure against metabolic syndrome; a study has shown association of higher intake of fruits and vegetables to a lowered risk of metabolic syndrome [48]. Furthermore, a cross-sectional survey on the prevalence of metabolic syndrome concerning leisure time physical activity on 60-year-old men and women highlighted the role of physical activity as a preventative measure for metabolic syndrome [49]. In addition, increased fluid intake was found to significantly reduce stone recurrences [45]. In a randomised trial, besides fluid intake, a low animal protein and low sodium diet combined with a high calcium diet was significantly better in prevention of recurrent stones, with recurrences nearly half that of the control group [46].

### 5.2. Secondary Prevention

Lifestyle change is also one of the most effective therapeutic interventions for metabolic syndrome, aside from being the primary prevention. An investigation, where obese individuals were enrolled in structured weight loss programme for one year, concluded that weight loss greatly improved all aspects of metabolic syndrome [47]. There is solid evidence that exercise and weight reduction improves insulin resistance in obese individuals by improving insulin sensitivity and endothelial function [50]. While there is no recommended diet for the individuals with metabolic syndrome, a Mediterranean diet appears to be beneficial due to its generally lower content of refined sugar and its high content of fibre, fruits, and vegetables [51]. According to the Oslo Diet and Exercise Study, the combination of both diet and exercise intervention was significantly more effective than just diet or exercise in the treatment of the metabolic syndrome [50].

Chronic dehydration decreases urine pH and increases urinary saturation of lithogenic factors both of which lead to crystal formation. Adequate hydration dilutes the urine and its supersaturation thereby delaying crystallisation. A randomised trial of studies [45] showed a beneficial effect of increased fluid intake and a reduction in animal protein and sodium but no additional benefit was demonstrated from dietary supplements. A fluid intake that maintains a urine output of >2 L is generally recommended in stone formers and although water has been recommended in most studies, beneficial effects have also been seen with lemon, cranberry, and apple juice [19]. A negative effect of soft drinks possibly due to an increase in acid load leading to a reduced pH was also seen. Citrate supplementation increases urinary citrate and pH thereby reducing stone formation. A nutritional approach tailored to the patient's risk factors for urolithiasis is perhaps the best way forward.

As of yet, there is no known single drug that effectively treats all components of metabolic syndrome equally. However, each component of metabolic syndrome can be treated independently with different types of drugs, including weight loss drugs, antidiabetics, antihypertensives, and antilipemic and anticoagulant drugs [52]. A practical ABCDE approach (see Table 3) provides a framework for recognising and implementing a management plan for metabolic syndrome in primary care [53]. It should however be mentioned that while metabolic syndrome may associate with urolithiasis, it has not been demonstrated that prevention methods for metabolic syndrome are effective for preventing stone disease; prevention procedures for kidney stones should be focused on treating the risk factors for stone disease in the individual patient [10, 36].

Highlighting the importance of prevention (primary or secondary), in addition to the physical health impacts on the patient from cardiovascular disease and other conditions, psychological conditions such as depression have also been found to be associated strongly with syndrome [54]. Furthermore, the economic drawback directly resulting from the condition has been estimated to increase medical costs by up to 24% [55].

## 6. Conclusion

The growing evidence of association between urolithiasis and metabolic syndrome suggests that urolithiasis may be a systemic disorder representing the interaction of multiple metabolic risk factors. With a rise in the incidence of both urolithiasis and metabolic syndrome and the cost associated with it, lifestyle modifications with dietary and preventative measure seem to be the most effective way to prevent primary and recurrent stone disease from a combined economic and patient wellbeing standpoint.

## Figures and Tables

**Table 1:** Definition of metabolic syndrome.

MetS component	Gender	IDF (2006)	AHA (2004)	NCEP ATP III (2001)	WHO (1999)	EGIR (1999)
Central obesity	Males	BMI > 30 kg/m^2^ and WC as per Table 2	WC > 102 cm	WC > 102 cm	WHR > 0.9 or BMI > 30 kg/m^2^	WC > 94 cm
	Females	BMI > 30 kg/m^2^ and WC as per Table 2	WC > 88 cm	WC > 88 cm	WHR > 0.85 or BMI > 30 kg/m^2^	WC > 80 cm

Raised triglycerides (TG)	Both	>150 mg/dL (1.7 mmol/L)	>150 mg/dL (1.7 mmol/L)	>150 mg/dL (1.7 mmol/L)	>1.695 mmol/L	>2.0 mmol/L

Reduced HDL cholesterol (HDL-C)	Males	<40 mg/dL (1.03 mmol/L)	<40 mg/dL (1.03 mmol/L)	<40 mg/dL	<0.9 mmol/L	<1.0 mmol/L
	Females	<50 mg/dL (1.29 mmol/L)	<50 mg/dL (1.29 mmol/L)	<50 mg/dL	<1.0 mmol/L	<1.0 mmol/L

Raised blood pressure (BP)	Both	Sys. > 130 mmHg or dia. > 85 mmHg or in treatment for HT	>130/85 mmHg or in treatment for HT	>130/85 mmHg or in treatment for HT	>140/90 mmHg	>140/90 mmHg or in treatment for HT

Raised fasting plasma glucose (FPG)	Both	>100 mg/dL (5.6 mmol/L)	>100 mg/dL (5.6 mmol/L)	>110 mg/dL (6.1 mmol/L)	—	>6.1 mmol/dL

Other	Both	—	—	—	Microalbuminuria (UAER > 20 *μ*g/min or ACR > 30 mg/g)	—

BMI: body mass index; WC: waist circumference; WHR: waist to hip ratio; HT: hypertension; UAER: urinary albumin excretion rate; ACR: albumin to creatinine ratio.

**Table 2:** Ethnic specific threshold guidelines for waist circumference.

Ethnic group	Gender	Waist circumference
Europids (in the USA, the ATP III guideline values are likely to be used for clinical purposes [males: 102 cm; females: 88 cm])	Male	>94 cm
	Female	>80 cm

South Asians (based on Chinese, Malay, and Asian-Indian population)	Male	>90 cm
	Female	>80 cm

Chinese	Male	>90 cm
	Female	>80 cm

Japanese	Male	>85 cm
	Female	>90 cm

Ethnic South and Central Americans	Use guidelines for South Asians until more specific data become available	

Sub-Saharan Africans	Use guidelines for Europids until more specific data become available	

Eastern Mediterranean and Middle Eastern (Arab) population	Use guidelines for Europids until more specific data become available	

**Table 3:** ABCDE approach to managing metabolic syndrome [52].

A	Aspirin		All patients with > 6% *10 year risk* (without contraindications)

B	Blood pressure control	Goal	<130/80 mmHg if at intermediate risk (>6% *10 year risk*)
		First line	ACEI or ACE
		Alternatives	*β*-blocker or diuretics may increase risk of diabetes

C	Cholesterol management		
	LDL-C	Goal	<130 mg/dL if at intermediate risk <100 mg/dL if at high risk
		First line	Statins
	Non-HDL-C	Goal	<160 mg/dL if at intermediate risk <130 mg/dL if at high risk
		First line	Statins, fenofibrate
		Alternatives	Omega-3 fatty acid supplement
	HDL-C		Long acting niacin may increase risk of glucose intolerance

D	Diabetes prevention	First line	Lifestyle modification
		Second line	Metformin, pioglitazone
	Diet		Weight loss, low glycemic load

E	Exercise		Daily moderate to rigorous exercise Recommend use of pedometer with goal of >10000 steps/day
